# Downregulation of miR‐133b predict progression and poor prognosis in patients with urothelial carcinoma of bladder

**DOI:** 10.1002/cam4.777

**Published:** 2016-06-12

**Authors:** Xiaonan Chen, Bin Wu, Zhenqun Xu, Shijie Li, Shutao Tan, Xuefeng Liu, Kefeng Wang

**Affiliations:** ^1^ Department of UrologyShengjing Hospital of China Medical UniversityShenyang110004China; ^2^ Division of Nephrology and Cancer CenterUniversity of CaliforniaDavisCA95616

**Keywords:** miR‐133b, prognosis, progression, urothelial carcinoma of the bladder

## Abstract

We found microRNA‐133b (miR‐133b) was downregulated in urothelial carcinoma of the bladder (UCB) tissues, and it could inhibit the proliferation and induce apoptosis in UCB cells. Consequently, we intend to explore the clinical significance of miR‐133b in UCB patients. Expression of miR‐133b in 146 UCB specimens and matched adjacent non‐neoplastic bladder tissues were measured by quantitative real‐time polymerase chain reaction. The overall survival (OS) curve and progression‐free survival (PFS) curve were plotted using the Kaplan–Meier method. Prognostic factors for OS and PFS were identified by univariate and multivariate analyses using the Cox proportional hazards regression model. The expression of miR‐133b was significantly downregulated in UCB tissues compared with those in adjacent non‐neoplastic bladder tissues (*P* < 0.001). Among UCB patients, low expression of miR‐133b significantly correlated with aggressive clinicopathological features. Multivariate analysis indicated that the expression of miR‐133b was the independent prognostic factors for predicting PFS (RR: 2.97; 95% CI: 1.78–6.44; *P* = 0.009) and OS (RR: 4.23; 95% CI: 1.51–11.8; *P* = 0.011) in patients with UCB. Our study demonstrated that downregulation of miR‐133b associated with aggressive clinicopathological features and predicted unfavorable prognosis in patients with UCB, might serve as feasible biomarker for clinical outcome of UCB patients after surgery and potential therapeutic target in the future.

## Introduction

Urothelial carcinoma of the bladder (UCB) is the most common urological malignancy in China, meanwhile it constitutes the second common urological malignancy in western countries. UCB usually accompanied with a high recurrence rate and mortality, about 50% of the UCB may relapse within 2 years after surgery 1. Although there have been many studies that showed some abnormal oncogene activation, the inactivation of tumor suppressor genes may play an important role in the tumorigenesis of UCB 2, 3, 4. But so far, the pathogenesis of UCB is not well understood, and there is no effective prognostic indicator yet. To improve the prognosis of UCB, new prognostic factor was highly wanted.

MicroRNAs (miRNAs) are a group of short, noncoding RNAs with 20–24 nucleotides length. Some studies indicated that miRNA was involved in the pathophysiology of many eukaryotes, which could regulate gene expression at the transcriptional level 5. More than 50% miRNA genes were located close to cancer‐related genes area, and researchers also identified many abnormal expression of miRNAs in various tumors. All of these suggested that such small nucleic acid molecules may play a critical role similar to oncogenes or tumor suppressor genes involved in a variety of human tumors 6. One certain miRNA can regulate the expression of multiple target genes, and a target gene may be regulated by many miRNA simultaneously. The complicated regulatory network was formed between miRNAs and their target genes 7. miR‐133b was first considered to be a muscle‐specific microRNA, however, some recent research revealed that there was significant correlation between abnormal expression of miR‐133b and tumorigenesis. miR‐133b may affect the ability of tumor cell proliferation, apoptosis, and invasion by regulating the expression of different target genes 8, 9. The abnormal expression of miRNA may act as a potential prognostic factor. Sun et al. reported that downregulation of miR‐126 may act as an independent negative prognostic factor of biochemical recurrence‐free survival in prostate cancer patients undergoing radical prostatectomy 10. Akçakaya et al. 11 found that combination of upregulation of miR‐185 and downregulation of miR‐133b could predict poor survival and higher metastatic probability. Recent study by Nofech‐Mozes et al. 12 suggested that renal cell carcinoma patients with higher miR‐194 expression has significantly longer disease‐free survival and overall survival compared to those with lower expression. Our previous studies had shown that miR‐133b could inhibit the proliferation of bladder cancer cells and induce apoptosis in bladder cancer cells, which indicated that miR‐133b play an important role in tumorigenesis and progression of UCB 13. We want to validate the importance of miR‐133b in UCB by analyzing the relationship between the expression of miR‐133b and clinicopathological characteristics of UCB.

## Materials and Methods

### Patients and specimens

Fresh UCB specimens and matched adjacent non‐neoplastic bladder tissues from 146 patients who underwent surgery between January 2008 and December 2010 at Shengjing Hospital of China Medical University were collected. All tissue samples were snap‐frozen in liquid nitrogen and then stored at −80°C for quantitative real‐time polymerase chain reaction (qRT‐PCR) assay. All specimens were pathologically re‐evaluated by two experienced pathologists to confirm the diagnosis of UBC. The pathological data were classified according to the 2009 UICC TNM classification for the stage and 2004 WHO classification for the grade. Papillary urothelial neoplasm of low malignant potential (PUNLMP) is a urothelial lesion that biologically carries a relative low risk of progression. But many of these patients had recurrences of up to 60% according to some studies 14. Some of these patients might progress to either low‐grade (LG) or high‐grade (HG) urothelial carcinoma 15. Considering relatively low but definitive risk of recurrence and progression, patients with PUNLMP should be treated similarly to patients with low‐grade, noninvasive urothelial carcinoma 16. Consequently, we enrolled the patients with PUNLMP in this study. None of patients who were included in this study had received radiotherapy or chemotherapy prior to surgical treatment. Informed consent was obtained from all study participants, and this study was approved by the Research Ethics Committee of Shengjing Hospital of China Medical University. A total of 94 male and 52 female were included in this study, the median age was 60 (range: 29–87) years. The median follow‐up period was 58 (range: 10–78) months. The clinicopathological feature of all patients is presented in Table 1.

**Table 1:** The relationship between miR‐133b expression and clinicopathological characteristics of bladder cancer

Clinicopathologic features	Cases*N* (%)	miR‐133b expression		*P*
		Low (%)	High (%)	
Gender				
Male	94 (64.4)	42 (44.7)	52 (55.3)	0.467
Female	52 (35.6)	20 (38.5)	32 (61.5)	
Age (years)				
<60	70 (47.9)	27 (38.6)	43 (61.4)	0.361
≥60	76 (52.1)	35 (46.1)	41 (53.9)	
ECOG				
≤1	127 (87.0)	49 (38.6)	78 (61.4)	0.014^a^
≥2	19 (13.0)	13 (68.4)	6 (31.6)	
T stage				
Ta	52 (35.6)	16 (30.8)	36 (69.2)	0.013^a^
T1	70 (47.9)	30 (42.9)	40 (57.1)	
≥T2	24 (16.4)	16 (66.7)	8 (33.3)	
Histological grade				
PUNLMP	33 (22.6)	8 (24.2)	25 (75.8)	<0.001^a^
LG	80 (54.8)	27 (33.8)	53 (66.3)	
HG	33 (22.6)	27 (81.8)	6 (18.2)	
Surgery				
Transurethral resection (TUR)	117 (80.1)	41 (35.0)	76 (65.0)	<0.001^a^
Cystectomy	29 (19.9)	21 (72.4)	8 (27.6)	
Recurrence or progression (postoperative)				
Yes	58 (39.7)	44 (75.9)	14 (24.1)	<0.001^a^
No	88 (60.3)	18 (20.5)	70 (79.5)	
Past history of urothelial carcinoma of the bladder (UCB)				
Yes	44 (30.1)	21 (47.7)	23 (52.3)	0.398
No	102 (69.9)	41 (40.2)	61 (59.8)	
Multifocality				
Single tumor	91 (62.3)	32 (23.8)	59 (76.3)	0.022^a^
Multiple tumor	55 (37.7)	30 (65.2)	25 (34.8)	
Death				
Yes	33 (22.6)	28 (84.8)	5 (15.2)	<0.001^a^
No	113 (77.4)	34 (30.1)	79 (69.9)	

PUNLMP, papillary urothelial neoplasm of low malignant potential; LG, low grade; HG, high grade; TUR, transurethral resection; USB, urothelial carcinoma of the bladder.

a indicates *P* < 0.05.

The medical records were reviewed retrospectively for demographics, performance status as defined by the Eastern Cooperative Oncology Group (ECOG) 17, histological grade, pathological stage, recurrence, progression, surgical procedure, solitary, or multiple lesions. Both overall survival (OS) and progression‐free survival (PFS) were analyzed in this study. The OS was evaluated from the date of surgery to the last follow‐up or death. The PFS was calculated from the date of initial surgery to the appearance of new local recurrence or distant metastasis.

### Total RNA extraction

Total RNA was isolated from frozen tissues using mirVana miRNA Isolation kit (Ambion, Austin, TX) according to the manufacturer's protocol. RNA concentrations and purity were measured using the NanoDrop ND‐2000 spectrophotometer (NanoDrop Technologies, Houston, TX). RNA samples with optical density A260/280 ratio close to 2.0 were subsequently utilized for reverse transcription.

### Quantitative real‐time PCR

Reverse transcription was performed according to PrimeScript^®^RT reagent kit protocol (Takara, Shiga, Japan). The specific complementary cDNA of miR‐133b was synthesized using gene‐specific stem‐loop primer (5′‐GTC GTA TCC AGT GCA GGG TCC GAG GTA TTC GCA CTG GAT ACG ACT AGC TGG TT‐3′), and the primer for U6 was 5′‐AAC GCT TCA CGA ATT TGC GT‐3′. Quantitative real‐time PCR was performed using the SYBR Premix Ex Taq^™^II (Takara, Shiga, Japan) on the 7500 Real‐time PCR System (Applied Biosystems, Foster, CA, USA). The qRT‐PCR primers were as follows: miR‐133b forward 5′‐GCG CTT TGG TCC CCT TC‐3′ and reverse 5′‐CAG TGC AGG GTC CGA GGT‐3′; U6 forward 5′‐CTC GCT TCG GCA GCA CA‐3′ and reverse 5′‐AAC GCT TCA CGA ATT TGC GT‐3′. The relative quantification of miR‐133b expression was calculated by the comparative cycle threshold (CT) method. U6 was used as an internal control to normalize the results. Each sample was tested in triplicate. To investigate the correlation between miR‐133b expression and oncological outcome, the patients were divided into two groups with high or low miR‐133b expression according to the median expression level.

### Statistic analysis

Continuous variables were expressed as means ± standard deviation (SD). Comparisons of miR‐133b expression between UBC tissues and adjacent non‐neoplastic bladder tissues were analyzed with Mann–Whitney *U* test. Chi‐square test was used to analyze the association between miR‐133b expression and the clinicopathological characteristics. The OS curve and PFS curve were plotted using the Kaplan–Meier method, and the differences were determined by the log‐rank test. Prognostic factors for OS and PFS were identified by univariate and multivariate analyses using the Cox proportional hazards regression model, and the risk ratio (RR) with 95% confidence intervals (CI) was calculated. Variables with significant values (*P* < 0.05) from univariate analyses were enrolled into the multivariate analyses. Differences were considered statistically significant at *P* < 0.05. All statistical analyses were performed using the SPSS software (version 22.0).

## Results

### miR‐133b is downregulated in UCB

**Figure 1:**
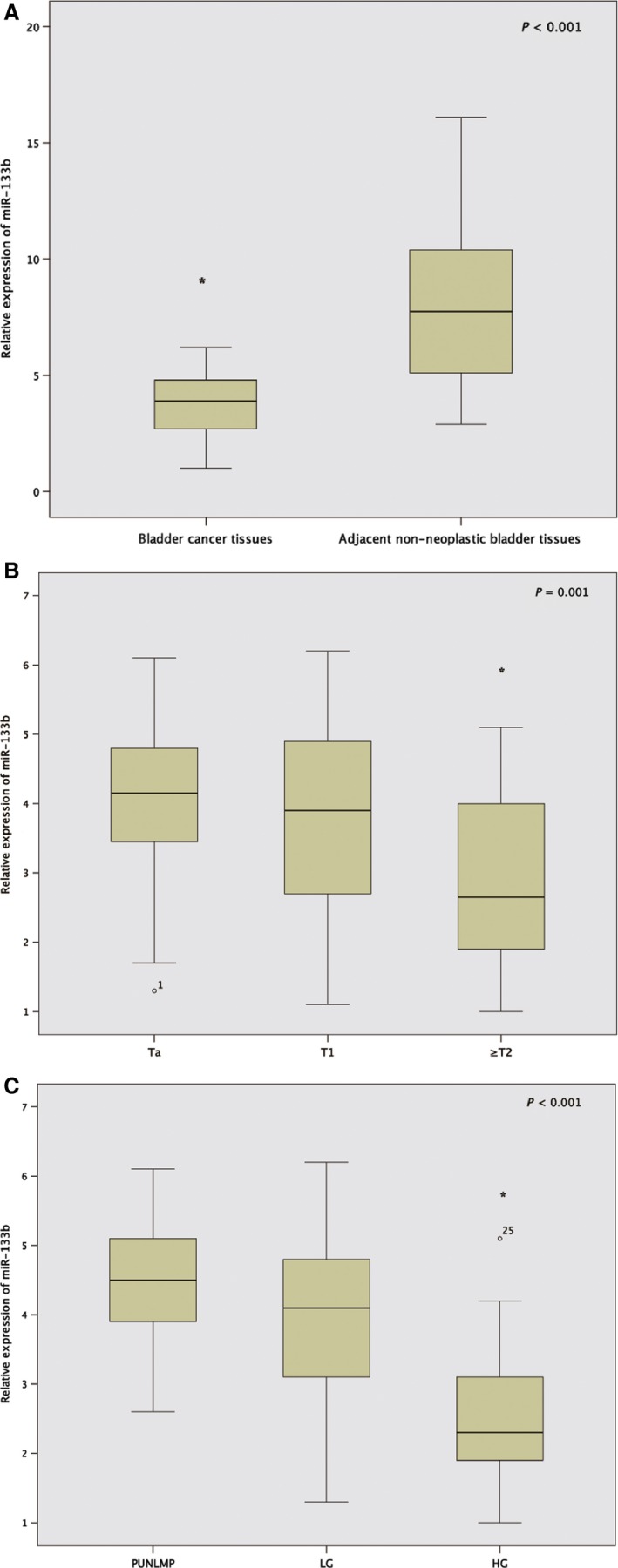
Expression of miR‐133b in 146 urothelial carcinoma of the bladder (UCB) specimens and matched adjacent non‐neoplastic bladder tissues were measured by quantitative real‐time polymerase chain reaction. (A) The expression of miR‐133b in UCB tissues and adjacent non‐neoplastic bladder tissues. (B) The expression level of miR‐133b in muscle invasive bladder cancer (≥T2) and nonmuscle invasive bladder cancer (Ta–T1). (C) The expression level of miR‐133b in UCB tissues with papillary urothelial neoplasm of low malignant potential (PUNLMP), low (LG) and high (HG) pathological grades, respectively. *indicates *P* < 0 .01.

The expression of miR‐133b was detected in 146 UCB specimens and matched adjacent non‐neoplastic bladder tissues, the results were normalized to U6. The expression of miR‐133b was significantly downregulated in UCB tissues compared with those in adjacent non‐neoplastic bladder tissues (3.74 ± 1.26 vs. 8.04 ± 3.32, *P* < 0.001, Fig. 1A). Among UCB tissues, we found the expression level of miR‐133b distinctly decreased in muscle invasive bladder cancer (≥T2) compared with nonmuscle invasive bladder cancer (Ta–T1) (Ta, T1 vs. ≥T2: 4.14 ± 1.02, 3.72 ± 1.29 vs. 2.98 ± 1.33; *P* = 0.001, Fig. 1B). The statistic results also showed that the expression of miR‐133b in both PUNLMP and LG bladder cancer were significantly higher than that in HG bladder cancer (PUNLMP, LG vs. HG: 4.43 ± 0.99, 3.95 ± 1.14 vs. 2.58 ± 1.03; *P* < 0.001, Fig. 1C).

### Downregulation of miR‐133b associates with aggressive clinicopathological features of UCB

We analyzed the correlation between the expression of miR‐133b and the clinicopathological features of UCB patients (Table 1). The mean expression of miR‐133b in UCB tissues (3.7) was termed as cutoff value to classify 146 patients to low‐expression group (*n* = 62) and high‐expression group (*n* = 84). We found there was significant correlation between low expression of miR‐133b and aggressive clinicopathological features, including advanced T stage (*P* = 0.013), high histological grade (*P* < 0.001), postoperative recurrence or progression (*P* < 0.001), and high mortality (*P* < 0.001). Interestingly, significant correlation was also found between the expression of miR‐133b and patients' ECOG score (*P* = 0.014), surgical procedure (*P* = 0.001), tumor multifocality (*P* = 0.22). However, the expression level of miR‐133b was not significantly associated with gender, age, and past history of UCB.

### Downregulation of miR‐133b predict poor prognosis in patients with UCB

**Figure 2:**
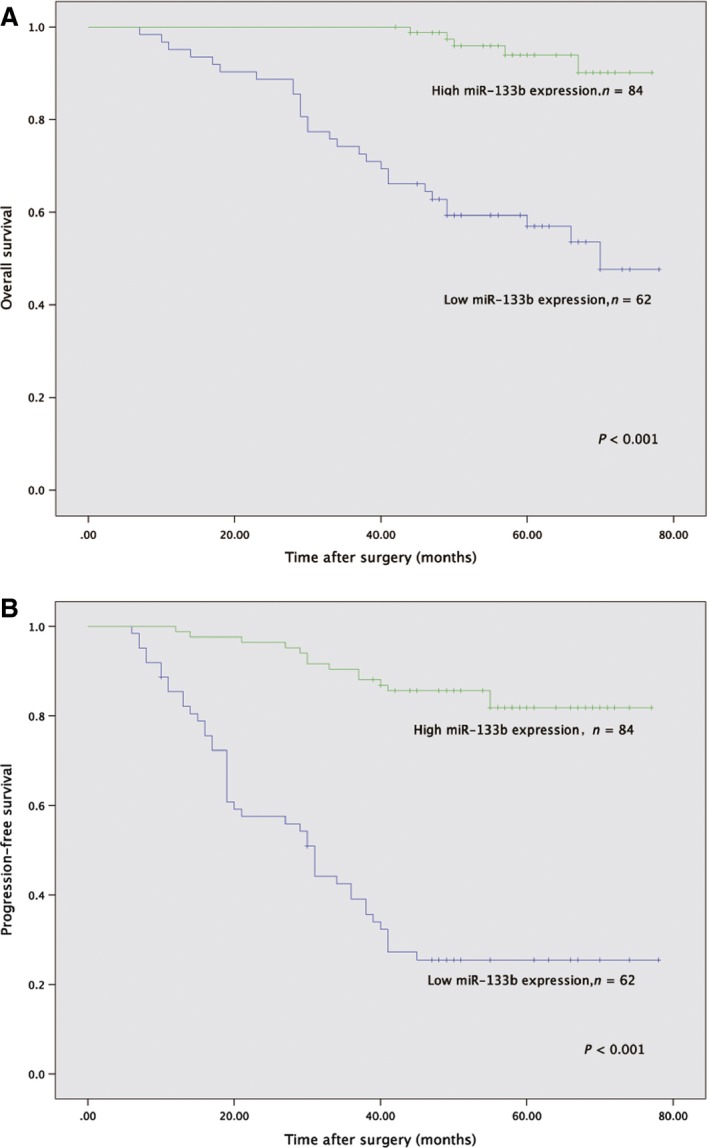
Kaplan–Meier curve for survival in patients with urothelial carcinoma of the bladder (UCB) according to expression level of miR‐133b. (A) Overall survival (low expression vs. high expression). (B) Progression‐free survival (low expression vs. high expression).

Moreover, we evaluated the prognostic value of miR‐133b expression in patients with UCB. Low expression of miR‐133b in UCB patients was significantly associated with unfavorable OS (*P* < 0.001, Fig. 2A). It was really clear that there was significant difference in PFS between low‐miR‐133b expression group and high‐miR‐133b expression group (*P* < 0.001, Fig. 2B). The 1‐, 3‐, and 5‐year postoperative PFS rates of patients with low expression of miR‐133b were only 85.4%, 42.4%, and 25.5%, respectively. In contrast, the 1‐, 3‐, and 5‐year postoperative PFS rates of high‐miR‐133b expression group were 97.6%, 90.5%, and 85.7%, respectively.

Univariate analysis showed ECOG score (*P *< 0.001), T stage (*P* < 0.001), tumor grade (*P* < 0.001), surgical procedure (*P* = 0.031), tumor multifocality (*P* = 0.003), and expression of miR‐133b (*P* < 0.001) were significantly associated with patient's OS. In terms of several prognostic factors, the following were significantly associated with PFS in UCB patients on univariate analysis: T stage (*P* = 0.010), tumor grade (*P* < 0.001), surgical procedure (*P* = 0.016), past history of UCB (*P* = 0.037), and expression of miR‐133b (*P* < 0.001).

Only the significant variables from univariate analysis were enrolled in the multivariate Cox regression analysis. Among these significant prognostic factors, multivariate analysis showed that ECOG score (RR: 2.59; 95% CI: 1.12–6.01; *P* = 0.026), T stage (RR: 2.42; 95% CI: 1.06–5.54; *P* = 0.037), tumor grade (RR: 3.55; 95% CI: 1.5–8.16; *P* = 0.013), and expression of miR‐133b (RR: 4.23; 95% CI: 1.51–11.8; *P* = 0.011) were the independent prognostic factors for OS in UCB patients. Furthermore, multivariate analysis also revealed that T stage (RR: 2.29, 95% CI: 1.25–4.61, *P* = 0.017), tumor grade (RR: 3.45; 95% CI: 1.84–7.12; *P* = 0.004), surgical procedure (RR: 2.02; 95% CI: 1.16–3.89; *P* = 0.041), and expression of miR‐133b (RR, 2.97; 95% CI: 1.78–6.44; *P* = 0.009) were the independent factors for predicting PFS in UCB patients. Detailed data of univariate and multivariate analysis were presented in Table 2.

**Table 2:** Univariate and multivariate Cox proportional hazard analyses for overall survival and progression‐free survival

	OS				PFS			
	Univariate		Multivariate		Univariate		Multivariate	
	RR (95% CI)	*P*	RR (95% CI)	*P*	RR (95% CI)	*P*	RR (95% CI)	*P*
Gender (male vs. female)	1.27 (0.60–2.67)	0.531			1.67 (0.91–3.08)	0.098		
Age, years (≥60 vs. >60)	1.96 (0.95–4.05)	0.068			1.69 (0.97–2.94)	0.064		
ECOG (≥2 vs. ≤1)	4.35 (1.99–9.51)	<0.001	2.59 (1.12–6.01)	0.026	1.49 (0.71–3.13)	0.289		
T stage (≥T2 vs. Ta–T1)	5.38 (2.69–10.79)	<0.001	2.42 (1.06–5.54)	0.037	2.46 (1.24–4.89)	0.010	2.29 (1.25–4.61)	0.017
Tumor grade (HG vs. PUNLMP~LG)	10.38 (5.0–21.55)	<0.001	3.55 (1.5–8.16)	0.013	3.74 (1.79–8.20)	<0.001	3.45 (1.84–7.12)	0.004
Surgery (cystectomy vs. TUR)	2.14 (1.12–3.97)	0.031	1.05 (0.66–1.97)	0.216	2.44 (1.29–4.62)	0.016	2.02 (1.16–3.89)	0.041
Past history of UCB (yes vs. no)	1.51 (0.76–3.08)	0.251			1.84 (1.03–3.27)	0.037	1.71 (0.98–3.02)	0.058
Multifocality (multiple vs. single)	3.01 (1.46–6.22)	0.003	0.93 (0.41–2.11)	0.859	1.04 (0.56–1.93)	0.912		
miR‐133b expression (low vs. high)	8.59 (3.69–15.86)	<0.001	4.23 (1.51–11.8)	0.011	3.36 (2.39–7.43)	<0.001	2.97 (1.78–6.44)	0.009

RR, risk ratio; CI, confidence intervals; ECOG, eastern cooperative oncology group; OS, overall survival; PFS, progression‐free survival; LG, low grade; HG, high grade; UCB, urothelial carcinoma of the bladder; PUNLMP, papillary urothelial neoplasm of low malignant potential; TUR, transurethral resection.

## Discussion

The morbidity of UCB had increased year by year. So far, surgery was still the main treatment strategy for UCB patients. But the postoperative recurrence rate is very high, and advanced UCB often accompanied with local invasion or distant metastasis, prognosis of advanced UCB was still unfavorable. We need to find more effective prognostic predictors, so that the UCB patients may receive personalized treatment according to their different prognosis.

Our study showed that miR‐133b is downregulated in UCB, which is consistent with previous study 18. Downregulation of miR‐133b had also been observed in other cancers, including colorectal cancer, gastric cancer, and renal cell carcinoma 19, 20, 21. Furthermore, we found downregulation of miR‐133b associates with aggressive clinicopathological features of UCB. Low expression of miR‐133b significantly associated with advanced T stage and high histological grade, which usually predicted poor prognosis. Consequently, we thought miR‐133b might become potential prognostic predictor. In addition, multivariate analysis showed that expression of miR‐133b was the independent prognostic factors for both OS and PFS in UCB patients. This is the first time to report that miR‐133b acts as a prognostic factor for UCB patients. Similarly, Chen et al. stated that low expression of miR‐126 and miR‐133b may play a crucial role in the progression and metastasis of non–small cell lung cancer, and downregulation of miR‐126 and miR‐133b correlated with shorter OS 22. Zhang et al. 23 found that downregulated serum level of miR‐133b and miR‐206 may predict poor prognosis in patients with osteosarcoma. Through comparing recurrent and nonrecurrent prostate cancer tissue samples, Karatas et al. 24 showed the expression of miR‐1 and miR‐133b in recurrent prostate cancer specimens significantly reduced, which may act as novel biomarker for predicting progression and recurrence of prostate cancer. Interestingly, our data illustrated that ECOG score was significantly associated with OS in UCB patients. Consistently, Hara et al. 25 had emphasized the usefulness of ECOG score in predicting clinical outcome after radical cystectomy for elderly patients. Moreover, multivariate analysis indicated surgical procedure was independent prognostic factor for PFS in UCB patient. Compared with patients under transurethral resection surgery, patients who received cystectomy appeared more prone to progress. There are several reasons behind this phenomenon, but we harbor the idea that the paramount reason is the patients with advanced UCB more likely to choose cystectomy. Hence, there were more advanced UCB patients in cystectomy group.

MircroRNA were involved in tumorigenesis by regulating some specific targeted genes. The mechanism for altered miR‐133b expression associating with prognosis of oncological patients had been revealed by many functional studies of miR‐133b. Our previous study demonstrated that miR‐133b could regulate proliferation and apoptosis of bladder cancer cells by targeting Bcl‐w and Akt1 13. Consistently, Zhou et al. 26 reported miR‐133a and miR‐133b may directly target the epidermal growth factor receptor and inhibit bladder cancer T24 and EJ cell's proliferation, migration, and invasion. In addition, Qiu et al. 27 stated that miR‐145, miR‐133a, and miR‐133b could inhibit proliferation, migration, invasion, and cell cycle progression of gastric cancer cells by regulating transcription factor Sp1. More recently, studies showed miR‐133b may affect the response of chemotherapy in certain cancer. Chen et al. 28 found miR‐133b significantly decreased in primary resistant ovarian carcinomas, compared with chemotherapy‐sensitive ovarian carcinomas. Their results indicated miR‐133b could downregulate drug‐resistance‐related genes (GST‐*π* and MDR1) and increase chemotherapy response of ovarian cell line to paclitaxel and cisplatin. Similarly, study by Chen et al. 29 manifested that concomitant downregulation of miR‐133a and miR‐133b may predict chemosensitivity of patients with esophageal squamous cell carcinoma undergoing paclitaxel‐based chemotherapy.

In conclusion, our study provides convincing evidence that miR‐133b is significantly downregulated in UCB patients. Downregulation of miR‐133b associated with aggressive clinicopathological features and predicted unfavorable prognosis, which indicates miR‐133b plays an important role in tumorigenesis of UCB. miR‐133b may serve as feasible biomarker for clinical outcome of UCB patients after surgery and potential therapeutic target in the future, although validation in further prospective studies is needed.

## Conflict of interest

None declared.

## Supporting information


**Table S1.** Relative expression of miR‐133b in 146 UCB tissues and matched non‐neoplastic bladder tissues.

**Figure S1.** Kaplan–Meier curve for survival in patients with invasive urothelial carcinoma of the bladder (UCB) according to expression level of miR‐133b. (A) Overall survival (low expression vs. high expression). (B) Progression‐free survival (low expression vs. high expression).
