# Supplementary material for: Downregulation of miR‐133b predict progression and poor prognosis in patients with urothelial carcinoma of bladder
Source: Cancer Med. 2016 Jun 12;5(8):1856–62. doi: 10.1002/cam4.777 (PMC4971914; doi:10.1002/cam4.777)
Supplement: Supplementary file 1 — Table S1. Relative expression of miR‐133b in 146 UCB tissues and matched non‐neoplastic bladder tissues. [file CAM4-5-1856-s001.docx]

| **Case** | **Age** | **Gender** | **Relative expression of miR-133b in UCB tissue** | **Relative expression of miR-133b in matched adjacent non-neoplastic bladder tissues** |
| --- | --- | --- | --- | --- |
| 1 | 57 | M | 2.2 | 5.6 |
| 2 | 79 | F | 3.1 | 5.8 |
| 3 | 57 | M | 2.9 | 13.3 |
| 4 | 83 | F | 2.6 | 7.5 |
| 5 | 76 | F | 1.8 | 7.1 |
| 6 | 73 | F | 2.9 | 5.1 |
| 7 | 85 | M | 2.1 | 12.1 |
| 8 | 71 | M | 1.9 | 3.9 |
| 9 | 57 | M | 1.9 | 5.5 |
| 10 | 62 | M | 1.3 | 9.7 |
| 11 | 70 | M | 1.3 | 7.1 |
| 12 | 63 | F | 4.9 | 5.3 |
| 13 | 55 | M | 4.7 | 3.6 |
| 14 | 49 | M | 3.9 | 6.1 |
| 15 | 68 | F | 5.1 | 8.1 |
| 16 | 60 | M | 5.1 | 5.5 |
| 17 | 46 | M | 5.1 | 4.6 |
| 18 | 52 | F | 2.1 | 9.4 |
| 19 | 47 | F | 3.1 | 4.5 |
| 20 | 41 | F | 3.9 | 7.1 |
| 21 | 57 | F | 6.1 | 10.5 |
| 22 | 60 | M | 5.1 | 15.1 |
| 23 | 59 | M | 3.7 | 5 |
| 24 | 42 | M | 3.8 | 9.1 |
| 25 | 75 | M | 4.7 | 10.6 |
| 26 | 63 | F | 4.9 | 2.9 |
| 27 | 66 | M | 3.9 | 9.8 |
| 28 | 67 | F | 3.1 | 10.7 |
| 29 | 73 | M | 2.2 | 8.1 |
| 30 | 79 | F | 5.1 | 3.1 |
| 31 | 75 | F | 5.1 | 4.1 |
| 32 | 72 | F | 4.1 | 9.9 |
| 33 | 75 | M | 1.9 | 9.1 |
| 34 | 49 | M | 4.1 | 11.4 |
| 35 | 50 | F | 4.2 | 3.6 |
| 36 | 63 | M | 3.1 | 4.1 |
| 37 | 81 | F | 4.6 | 14.5 |
| 38 | 69 | M | 3.9 | 11 |
| 39 | 29 | M | 4.4 | 16.1 |
| 40 | 61 | F | 5.8 | 8.1 |
| 41 | 74 | M | 4.1 | 9.2 |
| 42 | 70 | F | 3.5 | 8.1 |
| 43 | 52 | M | 4.8 | 13.1 |
| 44 | 48 | M | 3.9 | 11.1 |
| 45 | 53 | M | 6.1 | 6.4 |
| 46 | 46 | F | 4.1 | 3.1 |
| 47 | 61 | F | 4.8 | 14.1 |
| 48 | 51 | M | 5.1 | 4.1 |
| 49 | 63 | F | 4.1 | 4.9 |
| 50 | 58 | F | 4.9 | 9.1 |
| 51 | 47 | F | 4.8 | 7.7 |
| 52 | 61 | M | 5.6 | 5.3 |
| 53 | 52 | F | 6.1 | 4.1 |
| 54 | 71 | M | 4 | 11.9 |
| 55 | 75 | M | 3.7 | 7.1 |
| 56 | 43 | F | 5.5 | 5.8 |
| 57 | 51 | M | 2.7 | 5.3 |
| 58 | 43 | M | 5.1 | 11.6 |
| 59 | 71 | F | 3.6 | 10.4 |
| 60 | 65 | F | 4.9 | 8.1 |
| 61 | 61 | F | 2.1 | 10.1 |
| 62 | 39 | M | 5.6 | 12.1 |
| 63 | 55 | M | 2.7 | 7.8 |
| 64 | 60 | M | 1.7 | 8.1 |
| 65 | 47 | M | 5.9 | 5.4 |
| 66 | 46 | M | 2.2 | 5.1 |
| 67 | 63 | M | 2.1 | 6.8 |
| 68 | 57 | F | 2.5 | 7.4 |
| 69 | 47 | M | 4.7 | 4.9 |
| 70 | 59 | M | 4.1 | 3.8 |
| 71 | 47 | F | 2.9 | 8.9 |
| 72 | 64 | M | 4.8 | 13.1 |
| 73 | 68 | M | 2.5 | 8.9 |
| 74 | 44 | F | 3 | 7.1 |
| 75 | 81 | M | 2.7 | 5.1 |
| 76 | 37 | M | 4.4 | 4.1 |
| 77 | 50 | M | 3.9 | 4.1 |
| 78 | 68 | M | 4.1 | 14.4 |
| 79 | 66 | M | 5.2 | 6.9 |
| 80 | 70 | M | 5.1 | 13.1 |
| 81 | 66 | M | 4.2 | 10.1 |
| 82 | 68 | M | 4.1 | 15.1 |
| 83 | 52 | F | 4.8 | 4.4 |
| 84 | 61 | M | 1.9 | 7.9 |
| 85 | 58 | M | 4.2 | 16.1 |
| 86 | 37 | M | 5.1 | 13.2 |
| 87 | 62 | M | 5.2 | 13.1 |
| 88 | 70 | M | 4.9 | 8.9 |
| 89 | 76 | F | 2.6 | 9.1 |
| 90 | 39 | M | 2.1 | 6.1 |
| 91 | 57 | M | 3 | 9.8 |
| 92 | 64 | F | 5.5 | 15.6 |
| 93 | 52 | M | 3.1 | 3.9 |
| 94 | 50 | M | 2.5 | 11.2 |
| 95 | 67 | M | 4.5 | 6.1 |
| 96 | 70 | M | 3.9 | 4.1 |
| 97 | 40 | M | 4.9 | 7.1 |
| 98 | 37 | M | 4.4 | 13.1 |
| 99 | 50 | M | 1.5 | 10.1 |
| 100 | 69 | F | 3.3 | 3.9 |
| 101 | 68 | M | 4.8 | 13.1 |
| 102 | 65 | F | 3.4 | 3.9 |
| 103 | 78 | M | 1.5 | 8.2 |
| 104 | 87 | M | 1.9 | 6.4 |
| 105 | 64 | M | 1 | 6.6 |
| 106 | 68 | M | 2.1 | 8.3 |
| 107 | 52 | M | 1.4 | 7.1 |
| 108 | 37 | M | 1.7 | 5.9 |
| 109 | 70 | M | 2.5 | 6.7 |
| 110 | 57 | M | 2.8 | 5.1 |
| 111 | 84 | M | 1.1 | 10.4 |
| 112 | 77 | F | 2.3 | 4.1 |
| 113 | 64 | M | 5.6 | 5.9 |
| 114 | 57 | M | 3 | 4.9 |
| 115 | 57 | F | 2.9 | 6.1 |
| 116 | 55 | F | 4 | 9.1 |
| 117 | 77 | M | 3.1 | 9.8 |
| 118 | 38 | M | 5.1 | 5.6 |
| 119 | 58 | M | 3 | 6.8 |
| 120 | 64 | M | 5 | 6.1 |
| 121 | 38 | M | 4.7 | 10.6 |
| 122 | 45 | M | 5 | 9.2 |
| 123 | 56 | M | 4.1 | 4.2 |
| 124 | 41 | F | 3.1 | 9.8 |
| 125 | 68 | M | 6.2 | 4.1 |
| 126 | 61 | F | 3.1 | 8.2 |
| 127 | 56 | F | 4.1 | 4.9 |
| 128 | 58 | F | 3.9 | 12.1 |
| 129 | 67 | M | 4.1 | 16.1 |
| 130 | 67 | M | 3.1 | 5.6 |
| 131 | 82 | F | 5.6 | 12.1 |
| 132 | 59 | F | 4.2 | 10.6 |
| 133 | 41 | F | 4.9 | 9.8 |
| 134 | 58 | F | 3.9 | 11.9 |
| 135 | 66 | M | 3.9 | 9.1 |
| 136 | 63 | M | 2.7 | 3.8 |
| 137 | 42 | M | 2.1 | 4.1 |
| 138 | 73 | F | 4.1 | 10.9 |
| 139 | 51 | M | 2.2 | 10.4 |
| 140 | 51 | M | 4.2 | 7.9 |
| 141 | 68 | F | 4.1 | 6.9 |
| 142 | 68 | F | 4.4 | 8.1 |
| 143 | 40 | M | 4.9 | 4.1 |
| 144 | 52 | M | 3.9 | 5.1 |
| 145 | 63 | F | 3.9 | 4.7 |
| 146 | 53 | M | 3.3 | 9.1 |

Relative expression of miR-133b was normalized to U6 expression.
