# Supplementary figures and images for: Downregulation of miR‐133b predict progression and poor prognosis in patients with urothelial carcinoma of bladder
Source: Cancer Med. 2016 Jun 12;5(8):1856–62. doi: 10.1002/cam4.777 (PMC4971914; doi:10.1002/cam4.777)

Figure S1

A

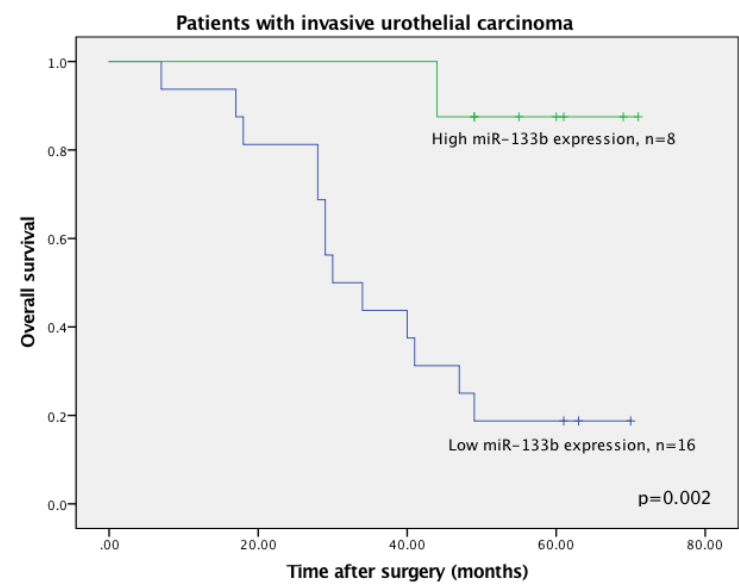

B

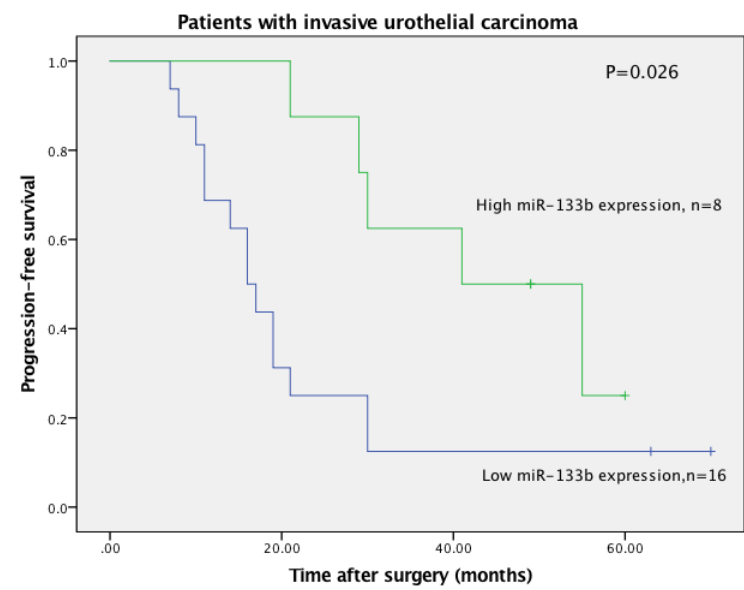

Supplement: Supplementary file 2 — Figure S1. Kaplan–Meier curve for survival in patients with invasive urothelial carcinoma of the bladder (UCB) according to expression level of miR‐133b. (A) Overall survival (low expression vs. high expression). (B) Progression‐free survival (low expression vs. high expression). [file CAM4-5-1856-s002.pdf]
